# Enhancing lupus outcomes by means of biology beyond overt clinical features, exemplified in an in-depth investigation of the effects of circadian rhythm disruption

**DOI:** 10.1136/lupus-2024-001215

**Published:** 2024-04-09

**Authors:** Ioannis Parodis

**Affiliations:** 1 Division of Rheumatology, Department of Medicine Solna, Karolinska Institutet and Karolinska University Hospital, Stockholm, Sweden; 2 Department of Rheumatology, Faculty of Medicine and Health, Örebro University, Örebro, Sweden

**Keywords:** Lupus Erythematosus, Systemic, Lupus Nephritis, Therapeutics, Antirheumatic Agents

## Introduction

SLE is a complex autoimmune disease, characterised by a prominent clinical heterogeneity,^1^ with lupus nephritis (LN) standing as one of its most severe manifestations.^2^ Despite significant advances in understanding SLE pathogenesis, the precise role of circadian rhythm disruption (CRD) in SLE and particularly its link to LN has remained elusive. In this issue of *Lupus Science and Medicine*, we are presented with a thought-provoking study by Shen *et al*, shedding new light on the intricate interplay between circadian rhythms and SLE severity.^3^

## Advancing the field

What sets this study apart is its comprehensive exploration of the impact of circadian rhythms on immune function and inflammation in the context of SLE. The authors embarked on a retrospective analysis, delving into clinical characteristics and transcriptional profiles of a large number of samples using advanced bioinformatics and machine learning methodologies. Through meticulous analysis, they uncovered compelling evidence of abnormalities within the circadian pathway in patients with SLE, indicating a potential link with the disease or disease states. Notably, the authors’ findings unveil an association between CRD and lupus flares, potentially implicating a role for CRD in disease evolution.

## Introducing the Flare Risk Score

Central to the authors’ investigation is the introduction of a Flare Risk Score (FRS), a tool designed to predict overall disease progression in patients with SLE. The FRS, developed through a rigorous analytical pipeline, exhibited good ability to forecast disease severity, offering a novel approach for patient stratification based on a risk profile. By incorporating both established parameters such as disease activity status and disease duration alongside new features of pathway enrichment, the FRS aspires to represent a more holistic view of disease prognosis.

## Insights into causality

Using Mendelian randomisation (MR), a powerful tool for inferring causality, the authors delved deeper into the potential relationship between CRD and SLE progression. The authors’ findings revealed an inverse causal association between CRD and SLE, suggesting that genetic predispositions to circadian rhythm disturbances may impact the risk of SLE. Furthermore, the study uncovered a causal relationship between CRD and glomerular disorders, shedding some more light on the intricate mechanisms underpinning renal involvement in SLE.

## Addressing criticism

The authors have taken into account criticism raised by reviewers, including concerns regarding the selection of datasets and the validation of the FRS. In their description of the MR algorithm that claims causality implications, the authors correctly mention the important principles that the genetic variants indexing the exposure must be (1) associated with the exposure (relevance), (2) independent of confounders of the exposure–outcome relationship (exchangeability) and (3) only associated with the outcome through the exposure (exclusion restriction). A major criticism concerned how these assumptions were met, especially the assumption of lack of confounding, given that the biological samples from which data were generated were collected in a cross-sectional fashion. The authors elegantly used longitudinal information from the time of diagnosis until the sampling occasion to build advanced predictive modelling constructs. The authors also provided explanations for their selective rather than broad approach for choosing among publicly available datasets, emphasising the need to minimise confounding variables and maximise dataset coherence.

Another important concern highlighted the complexity and heterogeneity of renal involvement in SLE, and challenged the authors with regard to what their models actually predict. Renal involvement in patients with SLE may comprise immune complex-mediated glomerulonephritis of proliferative type that is predominantly characterised by subendothelial immune deposits, glomerulonephritis of membranous type that is predominantly characterised by subepithelial immune deposits and intrarenal vascular lesions of thrombotic nature, among other conditions, which all are difficult to determine by routine clinical features and routine laboratory tests, making indispensable the performance of a kidney biopsy. Importantly, information on that level of granularity was lacking in the datasets used by the authors and was therefore not incorporated in the predictive models. While acknowledging limitations in available datasets, particularly regarding specific kidney tissue features, it is reassuring that the authors remain committed to refining their models and exploring avenues for validations, an anticipated question at issue in work to come.

## Implications for clinical practice

The authors claim that a compelling aspect of the study is its potential impact on clinical practice. By highlighting the significant association between CRD and SLE severity, the authors urge clinicians to consider incorporating circadian rhythm assessments into routine SLE management. This proactive approach could lead to tailored treatments upon informed selection that has incorporated CRD assessments, ultimately improving patient outcomes. Additionally, the authors advocate that FRS emerges as a promising predictive tool, offering a valuable resource for guiding patient monitoring and risk stratification. While such direct clinical implications will remain aspirational, the present investigation by Shen *et al* generates a foundation for more survey toward this direction in the future.

## Future directions

As with any innovative study, there are avenues for further exploration. The authors rightly acknowledge the heterogeneity of renal involvement in lupus, emphasising the need for future research to delve into specific types of kidney disease and their association with CRD. Incorporating kidney biopsy data and exploring additional datasets could enhance the accuracy and clinical utility of the proposed models. Moreover, ongoing efforts to refine the FRS and validate its predictive power will be crucial for its integration into clinical practice.

In conclusion, Shen *et al* have presented a compelling study that advances our understanding of CRD in the context of SLE. Their innovative approach, from the establishment of the FRS to the exploration of causal relationships using MR, opens new doors for personalised medicine in SLE management, a need that cannot be overstated in light of continuous advancements in the pharmacotherapy of SLE, introducing a paradigm shift from broad immunosuppression to targeted approaches and biology-informed decision-making.^4^ This need becomes even more apparent in light of only weak connections between immunological aberrations and organ manifestations in SLE.^5^

While challenges and limitations remain, studies like the one presented by Shen *et al* in the current issue of *Lupus Science and Medicine* serve as a beacon, guiding future research endeavours toward improved prognostication and tailored interventions for patients with SLE. As we continue to unravel the complexities of autoimmune diseases,^6^ such endeavours pave the way for a brighter future in lupus patient care.
